# The Cholera and Other Prevalent Diseases

**Published:** 1849-08

**Authors:** 


*The Cholera and other Prevalent Diseases.*—[The following remarks on the mortality and the state of the public health in London, during the last winter, while cholera prevailed in that city as an epidemic were read before the Westminster Medical Society, April 7th, by Dr. Webster. They are interesting at the present time, when that disease is prevalent in so many portions of our own country. It is hoped that the question of the effect of cholera on the general mortality of the places where it prevails, will not be wholly neglected among us.]

Having adverted to the recent sanitary movement, which must in time produce beneficial consequences, Dr. Webster said, so far from the fears entertained by timid persons, that the public health would materially suffer by the re-appearance of epidemic cholera, the aggregate number of deaths from all causes had considerably diminished throughout London during the last six months, in comparison with the same period of the previous year, particularly in regard to diseases of the respiratory organs, usually so prevalent and fatal in cold weather. In proof of this opinion, notwithstanding the extraordinary severity of scarlatina and presence of cholera, the gross mortality from all diseases, in London, during the last six months ending the 31st of March, was 30,263; whereas, during the parallel six months of the previous winter, the total amount rose to 36,060 deaths, being an excess of 5797, or 18.82 per cent. in favor of the current season. The author observed that the greatest difference occurred in diseases of the organs of respiration; by which, including influenza, in the winter of 1847 and 1848, the deaths were 11,197; whilst during the same six months, ending the 21st of March of the current year, only 5127 persons died from the same causes; being less than half the former amount, or an excess of 118.39 per cent. more deaths under this head, in the previous, than the winter just terminated. Dr. Webster then alluded to some of the pectoral diseases, and said, that 1965 persons had died from pneumonia this season, but 3159 the previous, thus giving an excess of 60.76 per cent. By bronchitis, 2047 died the last six months, whilst the number was 2984 in the former period, being 45.77 per cent. more than now. Again, 3040 died from consumption this season, against 3740 in the winter of 1847 and 1848, being nearly one quarter of the deaths greater from the same disease than recently. By influenza, only 78 deaths occurred, in place of 1739, registered during the former season. By measles, 391, instead of 1346, which thus caused two and a half times more deaths in the previous winter than the one just terminated. Scarlatina formed, however, a marked exception in respect of its virulence and mortality, having proved more fatal last winter than for many years; it was, in fact, the chief epidemic of the season; 2546 individuals, principally under 15 years of age, having died from that disease during the six months ending the 31st of March last, instead of 1362 during the parallel period of the previous year, although the mortality from the same cause was also then greater than ordinary. By typhus the deaths were fortunately less this year than last, 1585 having died from that cause during the present winter, in place of 2201 the previous, thus making an excess of 38.86 per hundred more in the last than in the present season. The author subsequently alluding to bowel complaints, remarked, contrary, perhaps, to expectation, that notwithstanding the existence of cholera, and the prevalent tendency to bowel complaints, diarrhea and dysentery had actually proved less fatal during the last six months than in the same period of the previous year. Thus, in the six months ending the 31st March, 1849, the deaths in London by diarrhea were 554, instead 644 in the same months of the year before. By dysentery, 135 then died, in place of 116 recently. This contrast is curious, seeing that cholera has prevailed more extensively than usual, by which epidemic malady, 984 persons died in London during the last six months; whereas, only 21 deaths are recorded in the previous winter. The author here observed that, great as the above amount of deaths by cholera may appear, it is not by any means so considerable as the mortality met with in the spring of 1832, when this malady also prevailed in London epidemically. For instance, in the month of March and the first week of April, of that year, as many persons died from cholera in the metropolis, during these five weeks, as throughout the entire six months ending ihe 31st March, 1849. Having now almost ceased to exist as an epidemic in London, as only four deaths had been recorded of cholera during the week terminating last Saturday, Dr. Webster believed little apprehension need be now entertained, although likely, as in the year 1832, the disease may again become epidemic next summer, or in the autumn when cholera usually prevails in this country, but, for the most part, of a mild and less fatal description. From the various data detailed to the Society, notwithstanding the prevalence of cholera, and the unusual mortality by scarlatina, the author considered London had not become by any means unhealthy, nor had the last winter proved insalubrious. Dr. Webster subsequently discussed the diathesis generally exhibited by the diseases now passed under review. Speaking generally, from his own observation, as likewise from the information of other practitioners, the author believed that almost every complaint recently met with assumed an asthenic character—if not at first, at least soon afterwards, and even in those instances of disease which are really inflammatory, they very often soon exhibited symptoms of great debility and exhaustion—similar, in fact, to the peculiarity noticed when the influenza was so prevalent last year in the metropolis. Scarlatina, measles, and diseases of the respiratory organs, come all within this category, and have required very different modes of management to the measures formerly found beneficial. Dr. Webster then adverted generally to the remedies employed, and the methods of cure recently adopted, which, he said, were generally tonic and stimulating. Exceptions might occur to this rule, but they were rare, even in those diseases of the chest which formerly required antiphlogistic treatment. The abstraction of blood appeared seldom, if ever, necessary, and it is now as uncommon to bleed any patient as it was formerly the reverse. Indeed, the lancet, like the sword of the soldier in the time of peace, might be said to have been laid up in ordinary. The author subsequently discussed the treatment pursued in the several diseases alluded to in his paper, which it is unnecessary now to particularize, as the plans adopted seemed generally based upon the symptoms manifested, and the principles he had enunciated. In concluding the communication read to the Society, Dr. Webster observed, that although scarlatina was really one of the most prominent and serious epidemics prevalent during the last six months, whereby nearly three times as many persons were carried off as by cholera, still the latter malady occupied by far the most public attention. With respect, however, to the management of scarlatina, the author said it differed essentially from the method other practitioners like himself had formerly found it expedient to employ. In previous epidemics of this eruptive disease, it was frequently necessary to resort to antiphlogistic measures, low diet, active purging, and even to bloodletting, either from the arm, or by leeches. During the recent epidemic, so far from depletion being required, or admissible, it was often advisable to commence supporting the system very early in the complaint, to give tonics, ammonia, wine, and sometimes even brandy, where the symptoms, apparently but not actually, seemed inflammatory, debility, depression, and a great want of tone in the system, being generally characteristic of the malady, whilst the remedies best adapted under such circumstances were of the above description. Fortunately, this severe complaint has recently considerably abated in virulence; and although still above the average of previous seasons, the consequent mortality is by no means now so great as it was about the latter part of last year, and the early portion of the current. However, at whatever period the present epidemic scarlatina may terminate, medical practitioners will not fail to remember its late great prevalence, rapid progress, marked symptoms of debility, and its unusually fatal character, as, likewise, the tonic stimulating plan of treatment which the disease almost invariably required.—*London Lancet.*

